# Exploring the usability of a smartphone application to monitor fatigue and activity for people with acquired brain injury

**DOI:** 10.1177/03080226231183293

**Published:** 2023-07-12

**Authors:** Leisle Ezekiel, Jose Juan Dominguez Veiga, Tomas Ward, Helen Dawes, Johnny Collett

**Affiliations:** 1 Centre for Centre for Movement, Occupational and Rehabilitation Sciences (MOReS), Department of Sports, Life Sciences and Social Work, Oxford Brookes University, Oxford, UK; 2 School of Health Sciences, University of Southampton, Southampton, UK; 3 The Insight SFI Research Centre for Data Analytics, Dublin City University, Dublin, Ireland; 4 Oxford Health Biomedical Research Centre, Oxford, UK; 5 College of Medicine and Health, Exeter University, Exeter, UK; 6 AIB Chair of Data Analytics at the School of Computing, Dublin City University, Dublin, Ireland

**Keywords:** Usability, brain injury, fatigue, ecological momentary assessment, mHealth

## Abstract

**Background:**

Fatigue after acquired brain injury (ABI) leads to detrimental changes in ABI survivors’ daily activities and participation. There is a need to capture individual’s experience of fatigue as it happens, to better support self-management of fatigue.

**Study aims:**

To investigate the usability of a real-time tracker of fatigue and activity (using ecological momentary assessment delivered by a smartphone application) and the feasibility of capturing activity and environmental factors using phone sensors.

**Methods:**

Participants wore an activity monitor and completed up to eight surveys a day on a smartphone app, for 6 days, completed the system usability scale (SUS) and were interviewed on their views of using the app. Interview transcripts were analysed using qualitative content analysis. Agreement between data from the phone’s sensors and the activity monitor was analysed using Kappa statistics.

**Results:**

Seven participants completed between 11 and 58 surveys. Mean score on the SUS indicated good perceived usability of the app. Phone sensors did not reliably capture physical activity or background noise. Participants found the app easy to use and perceived self-monitoring to help their understanding of fatigue. A fatigue-tracking app may be acceptable to ABI survivors and has potential to aid self-management of fatigue.

## Introduction

Persistent and disruptive fatigue is commonly experienced following acquired brain injury (ABI), with estimates of fatigue prevalence ranging from 27 to 73% (Acciarresi et al., 2014; Ponsford and Sinclair, 2014). Fatigue causes individuals to change their participation in daily occupations, particularly those of a social nature or those related to leisure and work (Palm et al., 2017; Törnbom et al., 2019). A survey of over 3000 ABI survivors in the United Kingdom (UK) revealed that 90% of respondents perceived fatigue to negatively affect their social life; 75% of respondents had an acquired brain injury for more than 2 years (Headway, 2019).

Current approaches to the management of fatigue within occupational therapy tend to rely on therapist’s observations or the patient’s recall of their experiences (Drummond et al., 2021). Such approaches are prone to cognitive biases as people try to recall and summarise their experiences and so may provide an incomplete or inaccurate picture of fatigue. Fatigue scales are also problematic, particularly where ratings of fatigue experience are completed retrospectively (Stull et al., 2009). For example, Heine et al. (2016) found that scores on retrospectively completed fatigue scales (such as the fatigue severity scale) were only weakly associated with how people with multiple sclerosis rate their fatigue in real time. Heine et al. (2016) suggest that conventional fatigue scales do not effectively assess people’s daily experience of fatigue. Hence decisions on how best to manage an individual’s fatigue are not based on sufficiently reliable information about the impact of fatigue on daily life.

One solution is to assess fatigue in real time using ecological momentary assessment (EMA). EMA consists of sampling an individual’s experiences and subjective states as they occur in real time within the individual’s natural environment (Shiffman et al., 2008). The repeated and longitudinal nature of EMA allows investigation of relationships between possible triggers of fatigue (such as types of activity), as well as the consequences of fatigue, thereby providing rich information to support the self-management of fatigue.

EMA is now predominately delivered using smartphone applications as smartphones are ubiquitous in the general population (Cornet and Holden, 2018), enable passive data collection through smartphone sensors, and have considerable data processing and data transfer capabilities (Heron and Smyth, 2010). Traditionally, notifications to complete an EMA survey are triggered at a fixed or random time points across the day but there is increasing interest in using smartphone sensors to trigger a survey. Sensor-triggered surveys enable investigation of individual experiences within pre-specified contexts, for example experiences of fatigue within noisy environments (Dunton et al., 2016).

However, EMA is not without its limitations. Mitchell et al.’s rapid review of EMA in monitoring outcomes following traumatic injury highlights how the type of technology used to deliver EMAs affects the quality of data, that EMA completion rates tend to reduce over time, and that intensive self-monitoring may affect participants behaviour and responses, a phenomenon known as reactivity (Mitchell et al., 2022). Optimising the usability of EMAs is therefore essential in optimising data quality and exploring users experiences of EMA may help to understand the potential effects of intensive self-monitoring.

Several studies evidence the feasibility of using EMA to monitor symptoms of people with neurological conditions (Forster et al., 2020; Juengst et al., 2017; Lenaert et al., 2020). They also demonstrate benefits such as finding differences in daily patterns of fatigue according to the sex and age of the participant, and between-person variability in the relationships between fatigue and activity (Lenaert et al., 2020b; Kratz et al., 2017). However, these studies relied on using self-reports of experiences to collect data rather than enhancing data collection with phone sensors, despite recommendations to triangulate self-report data with objective methods of data collection (Heron and Smyth, 2010).

Using an app to track symptoms and experiences (such as fatigue) in real time is potentially burdensome for users and so it is essential to understand factors that affect the usability of the app from the users’ perspective. Hence this study aimed to investigate the usability of a smartphone EMA app to track fatigue, activity and subjective energy in daily life, from the perspective of people with ABI. A secondary aim was to investigate the feasibility of using phone sensors to capture activity and environmental factors linked to fatigue.

For the purposes of this study, usability was defined as the extent and ease of which specified users can use the app in the context of their daily lives and includes dimensions of efficiency, effectiveness and satisfaction (Coursaris and Kim, 2011). Fatigue was defined as ‘subjective awareness of a negative balance between available energy and the mental and physical requirements of activities’ (Cantor et al., 2014: 491), where energy captured the feeling of having capacity to complete mental or physical activities (Puetz, 2012).

Several qualitative studies exploring fatigue report that ABI survivors perceive daily activities and environmental factors (such as noise) to trigger their fatigue and suggest that understanding triggers and interactions between activities, energy and fatigue levels forms an essential part of managing fatigue (Ezekiel et al, 2020; Theadom et al., 2016).

## Materials and methods

The study was approved by the Oxford Brookes University Ethics Committee. The study was completed as part of a PhD programme, and data collection was conducted between December 2018 and August 2019.

### The application

We developed an Android phone application (app) which notified the app user throughout the day to complete a short survey about what they were doing and their subjective energy levels and prompted them to complete a short reaction time test. A self-report of energy was chosen because both people with ABI and occupational therapists use the concept of energy in fatigue management and because it reflects an asset-based approach to managing fatigue (Ezekiel et al., 2020; Malley et al., 2014).

A reaction time test was chosen as a potential objective indicator of fatigue. Reaction time tests are used to investigate the effects of sleep loss in healthy individuals but have been suggested as an objective proxy for fatigue in individuals with brain injury (Price et al., 2017; Sinclair et al., 2013).

**Figure 1.**
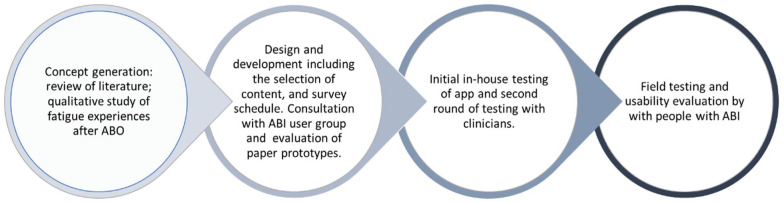
User-centred design of the application.

Background noise in decibels was detected through the phone sensors (Figure 1). In the reaction time tests, participants were asked to touch the phone screen as soon as the stimulus appeared. The stimulus was presented 20 times, with 5 stimuli randomly presented in each 30-second block.

**Figure 2.**
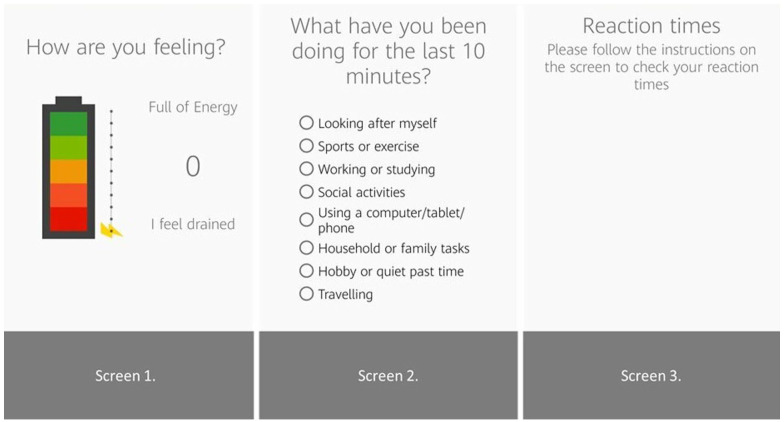
Application screens.

The app was developed following an iterative user-centred design process involving people with brain injury and occupational therapists. For example, following feedback about the activity question in the EMA, the available responses branched out to more detailed activities, allowing more choice when answering the question ‘what have you been doing for the last 10 minutes?’ (Figure 2). See Figure 1 for an outline of the process.

### Participants and setting

Participants were recruited through online advertising with support group for people with ABI and a UK-based charity for stroke survivors. ABI survivors were eligible to participate in the study if they reported having ABI-related fatigue, were able to use an Android smartphone, were able to give informed consent and were able to communicate sufficiently in English to follow instructions and participate in an interview. The ability to give informed consent was determined by the lead researcher through conversations about the study. None of the participants had prior involvement in developing the app. The study was conducted remotely using postal services and videoconferencing.

### Data collection

After giving informed consent, participants completed a short demographic questionnaire, then wore an activity monitor (Axivity 3: https://axivity.com/) on their wrist for 6 days whilst they field tested the fatigue app. Participants without access to an Android phone were loaned an Android phone for 1 week, with the app installed. Data on subjective energy levels, daily activity and reaction times were collected through the app. Reaction times greater than 350 ms were counted as lapses in concentration (Basner and Rubinstein, 2011). Data on the participants’ physical activity levels and background noise (in decibels) were collected through the smartphone sensors. Data on physical activity transitions were collected through the activity monitor. The activity monitor data were analysed in 60-second periods using OMGUI software, v1.0.0.37, Axivity. The programme then calculated whether periods were spent in sedentary, light, moderate and vigorous activity by applying ‘cut-points’ that correspond to different intensities of activities (Esliger et al., 2011).

Participants were notified to complete a survey eight times a day for 6 days. The survey schedule consisted of two fixed-time notifications and six random stratified notifications occurring between 10 am and 8 pm, with the option for users to additionally self-trigger assessments. The random notifications were stratified from 11 am to 7 pm, with a minimum of 60 min between notifications.

Following field testing, participants completed the system usability scale (SUS), a 10-item questionnaire that provides a global view of perceived usability (perceived effectiveness, efficiency and satisfaction) and has high internal consistency and satisfactory reliability (Brooke, 2013; Lewis, 2018).

Participants attended an online interview where the lead researcher used a semi-structured interview guide to explore participants’ experiences of using the app. The interview guide enabled exploration of participants’ experiences of self-rating their energy, completing the reaction time test, as well as their views on ease of use and satisfaction with the app. All interviews were audio-recorded and later transcribed.

### Analysis

The interview transcripts were analysed by the lead author (LE) using inductive content analysis to identify common factors affecting participant’s usability of the app. LE took an essentialist approach to the interview data where it is assumed that peoples’ descriptions of their experiences are an accurate reflection of their experience (Sandelowski, 2010). The data analysis was reviewed by members of the research team and an audit trail of analysis decisions was kept, enhancing transparency of the analytical process. The SUS was summarised using mean and standard deviation.

To investigate the feasibility of using phone sensors to capture activity and environmental factors linked to fatigue, the agreement between activity transitions (as recorded by the app using phone sensor data) and activity levels (as recorded by AX3) was investigated for each user. Axivity 3 data were analysed in periods of 60 seconds using OMGUI software, v1.0.0.37, Axivity. Activity transition (as detected by phone sensors) and activity levels recorded by the AX3 were categorised as ‘still’ or ‘movement’ and agreement between the phone and AX3 data were then assessed using Kappa statistics (McHugh, 2012).

For each participant, sampled levels of noise in decibels were inspected, and the number of instances recorded where noise levels were above 45 decibels was detected.

Participants’ responses to app notifications and completion rates were counted. Participants’ ratings of energy and reaction times are not presented here because usability issues would affect data quality.

## Results

Seven people with ABI agreed to take part in the study (see Table 1 for participant details). Participants’ ages ranged from 37 to 73. Only two participants were able to use their phone for the study, and the others carried a second (study) phone with them. Participants reported use of apps ranged from not using apps to using apps to assist them in daily life.

**Table 1.** Summary description of participants with ABI.

Participant ID	Age	Gender	Time since brain injury (years)	Used own or study phone	Usual app use on phones
1	61	Man	6	Study phone	Uses apps to assist in daily life and for entertainment purposes
2	71	Man	9	Study phone	Uses apps for entertainment purposes only
3	37	Woman	2	Study phone	Uses apps to assist in daily life
4	72	Man	2	Own phone	Uses apps to assist in daily life
5	47	Man	32	Study phone	Does not use apps to assist in daily life or entertainment purposes
6	51	Man	3	Own phone	Uses apps to assist in daily life and for entertainment purposes
7	73	Woman	2	Study phone	Uses apps to assist in daily life

### Technical effectiveness of the app

Technical effectiveness was assessed through examining the survey completion rates and by comparing data collected from the app with data collected from the activity monitor.

Participants completed between 11 and 58 surveys whilst field testing the app (Table 2). Participants were able to self-trigger an EMA and several tended to self-report rather than respond to a trigger. Participants rarely used the option to skip a survey. With regards to notifications sent at a fixed time (i.e. 10 am and 8 pm), the response rate was poor with five out of seven participants responding to less than half of the fixed-time notifications.

**Table 2.** Details of completion rates to EMA notifications.

Participant	Total no. of notifications sent by the app	Total no. of surveys completed	No. of completed surveys that were self-triggered	No. of responses to 12 fixed-time notifications
1	48	19	9	3
2	48	48	1	8
3	48	24	11	2
4	50	58	8	12
5	48	30	27	0
6	40	11	8	1
7	48	36	0	4

Six participants completed the SUS, and from these, the mean score was 82.5 (maximum score of 100), range 62.5–92.5, with only one participant scoring less than 68. One participant did not return the completed SUS. This score is interpreted as the app having a good level of usability and places it in the top 90–05 percentile of rankings of mean SUS scores (Bangor et al., 2008).

### Comparison of app data (activity transitions) and physical activity data from the activity monitor

Wear time of the AX3 ranged from 63% to 100% during the hours the EMA sampled (10 am–8 pm), with five participants wearing the AX3 for 90% of the time or more. Axivity data were lost for participant 5 because of an error in the activity transition file. The activity transitions recorded by the phone sensors for participants 4, 6 and 7 indicated extended periods where the phone was stationary and so was unlikely to reflect the participant’s movements.

The Kappa statistic was completed for the remaining three participants and indicated fair agreement for three participants (Kappa statistic of 0.412, 95% confidence interval: 0.156–0.667; Kappa: 0.315, 95% confidence interval: 0.128–0.503) and not statistically significant for one participant (Landis and Koch, 1977).

### Sampling of noise data

The level of noise detected by each of the phones’ sensors was low, ranging from silence to a conversational speech. Participants 3, 5 and 6 reported keeping their phone in a bag, which would have limited the phone sensor’s detection of noise.

### Findings from the content analysis of interviews with ABI survivors

Content of the interview transcripts was coded and grouped into four main categories: participants’ perspectives on using the app; participants’ reactions to self-monitoring; barriers to using the app and perspectives on future developments.

#### Participants’ perspectives on using the app

All of the participants identified aspects of the app that they liked. Three participants liked the options of delaying their response or ignoring it when notifications were at an inconvenient time. Participant 3 commented on how ‘normal’ it is to use an app, so found it to be socially acceptable. Three participants reported finding the app was easy to learn and easy to use.

Participant 2: ‘*it’s well balanced and not complicated, which I think is very important for those of us that are struggling with fatigue’.* (man, aged 71). The frequency of notifications was reported to be manageable for participants but two found the fixed alert times didn’t match their lifestyle and would have liked the option to change the times.

The numeric rating scale of energy was reported as a positive feature of the app by four of the participants. They also found the analogy of a battery and changing energy levels related to their experience of fatigue.

Participant’s experience in answering the activity question was more variable, with some reported the activity categories to be too restrictive, whilst others thought the categories were comprehensive. One participant struggled to categorise activities that served two purposes. He felt that the way his brain injury affected his thinking made answering this question more difficult, particularly if no category directly matched his activity.

Participant 1: the thing is gym work, I think. Cause that’s kind of like socialising for me. I get everything compartmentalised is what I’m trying to say so a bit more specific (man, 61).

Selecting the activity that most closely matched what participants were doing was further hampered by not having a ‘back button’ on the activity question. This meant that participants had to remember where activity options were located. Participant 2 thought it was helpful to stop him from ‘getting stuck in a loop’ whilst trying to decide what to choose, whereas other participants became ‘stuck’ on the wrong subcategory of activities.

With the reaction time test, participants described completing the test both as a game and as a potential ‘chore’. Two participants reported that the length of the test was just about acceptable for a short period but that they would not have tolerated a longer test. Participant 7 suggested either shortening the reaction time test or completing it less frequently. Others became competitive and tried to beat their score. Several perceived their reaction times to be faster when they felt alert or noticed that they were less able to concentrate on the test.

Participant 3: I guess it was just quite illuminating how many different seconds there is between when you feel energised or when you feel sleepy or how that doziness does affect your concentration (woman, aged 37).

### Participants’ reactions to self-monitoring

Four participants reflected on how repeatedly answering questions about their activity and energy started to change their understanding of fatigue or their behaviour in coping with fatigue.

Participant 1 noticed the impact of his sleeping patterns on his daily energy levels and decided to change his approach towards managing his sleep. Participant 2 explained that he started to think differently about his fatigue and questioned whether he needed to rest or whether he had enough energy to do something else.

Participant 2: Well it just made me do it more often than I normally do whereas – but in a normal day, I wouldn’t have been thinking ‘are my energy levels at above five or below five’. I would just be thinking ‘well I’m feeling alright or I’m not’ . . .I suppose what it did was made me think about how much – how I felt not just ‘oh it’s time to go and have a rest (man, aged 71).

Four participants commented that thinking about energy rather than fatigue was more positive as the concept of energy ‘encapsulates more’ than fatigue and prompted participants to consider their energy as a resource for participating in daily activities.

#### Barriers to using the app

Four participants identified two key barriers to using the app. The first was having two mobile phones, for those who used the study phone. One participant found carrying two phones ‘confusing’ (participant 1), whilst several others kept their phone in a bag and were aware of missing notifications (participants 3–6). The sound notification was the other key barrier to using the app as participants reported missing surveys because the notification was too quiet.

#### Perspectives on future developments

Four participants were interested in the apps’ potential to predict their fatigue, notify them of when to rest and capture their experiences over time to see how their fatigue changed. Participant 1 wanted to capture information about their sleep (both at night and in the day) to help manage their fatigue. Six participants wanted to personalise the app so they could change the alert to make it more noticeable, adjust fixed-time notifications to match their routine or amend activity categories and better reflect individual’s lifestyles.

## Discussion

In this study participants positively evaluated tracking their fatigue in real time and perceived the app as easy to use and easy to learn. They also perceived the app to be useful as it related to their experience of fatigue and activity. Additionally, the findings suggests that the act of structured self-monitoring influenced participants’ behaviour as they started to evaluate their experience of fatigue and activity. Repeated completion of an objective indicator of fatigue (short reaction time test) was perceived to be useful by participants but was also seen as onerous at times.

Several factors adversely affected the usability of the app from the participants perspective, and these were mostly linked to how the user was notified of an alert (thereby affecting the completion rate of EMA’s) and the cognitive load of the activity question (affecting the accuracy of responses). Using two phones also affected the study outcomes, particularly the effective use of passive sensor data to augment the fatigue app.

Participants’ perceived usability of the app (as rated by the SUS) was high (Bangor et al., 2008). A high mean SUS score does not assure the success of the app but it is a positive indicator of potential success, whereas low SUS scores indicate serious usability problems which are likely to limit the use and acceptability of the app (Bangor et al., 2008). Hence the participant’s perceived usability of the fatigue app is promising in this initial round of development.

From the content analysis, ease of use and learnability were identified as contributing to the usability of the app. It is likely that the simplicity of instructions, design interface and linear progression through the app were key to optimising the usability of the app. Ease of use and learnability are both key dimensions of usability but are particularly important when considering use by people with ABI and fatigue. A qualitative study by Engstrom and colleagues explored ABI survivor’s experience of using everyday technology, including the use of mobile phones. Their participants reported increased difficulty in learning new technology and following instructions after their brain injury (Engström et al., 2010). Fatigue was also cited as a barrier to engaging with technology. Hence ease of use and simple instructions are essential to usability for people with brain injury as they reduce the cognitive load of using the app.

From the content analysis, participants’ perceived usefulness of the app most likely contributed to the number of EMAs completed even though the phone notification was difficult to hear. This finding is in line with Ancker et al.’s qualitative study of individuals’ perspectives on tracking their health data using diaries (Ancker et al., 2015). Ancker and colleagues found that participants kept personal health data to help them make sense of their symptoms or to track their progress. However, participants became disillusioned with tracking if they could not connect their behaviour and their health data. Hence the usefulness of information collected is a key motivator for individuals tracking their health condition. It seems that the concept of battery recharging or draining resonated with participants and contributed to their perceived usefulness of the app. Perceived usefulness is a key factor affecting peoples’ uptake and use of technology even when usability issues affect the users’ experience (Lin, 2013).

The act of self-monitoring in fatigue in real time seemingly influenced participants understanding of fatigue as they developed explanations of situational factors affecting their fatigue, despite receiving no feedback from the app. Self-monitoring is a key behaviour change strategy and a core part of self-management so it is unsurprising that our findings point to evidence of reactivity to the EMA (Mohr et al., 2014). As individuals monitor their experiences (in this case, fatigue and activity), they increase their awareness of potential triggers, behaviours and patterns of experience, and this increased awareness brings about changes in behaviour. Health professionals also explicitly use self-monitoring of fatigue and activity tracking (recorded in a daily diary) as part of fatigue management interventions (Drummond et al., 2021). The advantage of EMA over paper-based diaries is that EMA reduces the impact of cognitive bias and leads to a more data-driven understanding of fatigue. However, our findings highlight the tension between EMA as assessment and EMA as a brief intervention; the effect of EMA on behaviour means the data collected may not be generalised to times when the individual is not tracking their fatigue. Further research is needed to evaluate how self-monitoring fatigue in real time affects individuals fatigue and fatigue-related behaviour (Lenaert et al., 2020).

Participants reacted positively to monitoring their energy levels as opposed to fatigue, potentially reflecting a strengths-based approach as they contemplated their available energy alongside their planned activity (Ezekiel et al., 2020). The concept of conserving energy to manage fatigue is used by occupational therapists across a range of health conditions, and there is moderate evidence that implementing energy conservation principles reduces the impact of fatigue on daily life in other health conditions (Blikman et al., 2013; Bennett et al., 2016).

However, it is important to note that whilst fatigue and energy are closely related constructs, they may not be polar opposites of the same construct (Loy et al., 2018). Associations between self-reports of energy and fatigue in other health conditions have been reported elsewhere (Braley et al., 2012) but a narrative review by Loy and colleagues suggest that fatigue and energy are independent constructs and may even reflect different physiological processes. Within the current study, it was assumed that fatigue is experienced when energy levels are low, reflecting the hypothesis that fatigue is triggered when energy resources are low (Loy et al., 2018). However, further research is needed to test the relationship between fatigue and energy.

### Factors limiting the effectiveness of the fatigue app

Data from qualitative interviews and the low completion rates of fixed and random notifications suggest the lack of personalisation of the app, and the design of the activity question affected its usability. It seems likely that personalising the app so that participants could adjust the timings of the time contingent surveys would improve usability.

Low completion rate of surveys triggered by the app and high numbers of self-triggered surveys may lead to over-representation of specific experiences. For example, the user may be more likely to trigger a survey when they feel fatigued, thus over-representing their experience of fatigue. For the fatigue app to be effective, it needs to sample a representative range of activities and experiences. Further work is needed to ensure the app samples to provide coverage of experience across the day.

From the qualitative data, the design of the activity question increased the cognitive load of using the app and limited the accuracy of the data collected. Kettlewell’s stakeholder evaluation of a smartphone app developed for people with ABI similarly identified a need to account for user’s cognitive problems when developing apps (Kettlewell et al., 2018). The design of the multiple-choice activity question needs revising to avoid relying on memory as to what activity is in which category.

Data from the phone sensors to detect noise and activity transitions were ineffective because of how the phone was carried and the use of a study phone. Whilst there has been considerable research investigating the accuracy of activity recognition by phone sensors, the studies depend on users carrying the phone on their person, but this does not reflect real-life use of smartphones. (Case et al., 2015). For example, in our study, participants reported keeping the phone in a bag when they were out of the house, thereby affecting the detectable noise levels. These findings do not preclude using phone sensors to augment the fatigue app but suggest further testing is needed to develop algorithms for sampling sensor-based events.

### Strengths and limitations

The focus on ABI survivors’ experiences of field testing the app was a key strength to the study as it provided insights into the perceived usefulness of the app and the potential burden of the EMA schedule as participants went about daily life.

Whilst this evaluation identified several usability issues, the lack of objective measures of usability means that the frequency with which usability issues occurred was not established. Including an automated evaluation tool would enable tracking of participants interactions with the activity question and help to quantify how often participants struggled with the question and which categories were problematic.

Finally, offering participant’s use of a study phone meant the recruitment process was more inclusive but negatively affected the EMA completion rate as participants then carried two phones with them. Installing the app onto participants own phones is likely to increase ecological validity because the participant’s phone use is closer to their usual behaviour.

### Recommendations

This usability evaluation highlighted the need to understand how brain injury affects peoples’ engagement and use of apps. Allowing personalisation of the fatigue app would also enable a better fit with users’ lifestyles and needs.

Further research is also needed to establish the functionality of sensor-based event sampling for this population. Combining data from multiple phone sensors may be more effective for defining and sampling events where the participant is in a highly fatiguing environment. Pairing the app with a wearable activity monitor would also provide rich data about participants’ physical activity levels and sleep duration, both of which are relevant to consider when managing fatigue.

Tracking fatigue and activity in real time provides large amounts of data which then needs to be presented in an accessible and useful format to the person with brain injury. Hence individualised feedback should be co-designed with people with brain injury to ensure that it is accessible and useful to end-users. Additionally, a data-driven approach to understanding an individual’s fatigue, as it happens in daily life, supports the development of more personalised and nuanced fatigue management strategies.

## Conclusions

This results of this user-centred enquiry into the usability of a real-time assessment of fatigue identified ease of use and learnability contributed to its usability, whereas lack of personalisation limited the effectiveness of the app. The findings also point to potential therapeutic benefits of self-monitoring fatigue using a smartphone app. Furthermore, the study highlights the need to consider a participant’s habits in how they carry the phone when using such technology. Individual’s phone-use behaviours limit the feasibility of using phone sensor data for event contingent EMA’s. Further refinement and development of the fatigue app is recommended to increase both its usability and usefulness in contributing to self-management of fatigue.

Whilst a main strength of the study was its user-centred approach to evaluating usability, the lack of objective measures of usability means the frequency and size of each usability problem cannot be determined. Nonetheless, this study highlights the importance of user-centred usability evaluation in the development of symptom-tracking apps and in optimising the acceptance of such apps by end-users.

**Key findings**

The process of monitoring subjective fatigue and activity in real time has potential to increase our understanding of fatigue and daily situational factors affecting fatigue and to support collaborative discussions between therapists and their service users.

Individuals’ phone-related behaviours limit the efficacy of using phone sensors to capture contextual information about the persons’ activity or environment.

**What the study has added**

This study suggests that self-monitoring fatigue in real time may affect ABI survivors understanding of fatigue, particularly relationships between fatigue experiences and activity-related behaviour.
